# Amputations

**Published:** 1918-08

**Authors:** 


**Report on Amputations of Inter-Allied Conference on the Care of Disabled Sailors and Soldiers.** *The Lancet,* p. 881, June 22, 1918.

A. The *kinematic operation* of Vanghetti was discussed by Prof. V. Putti (Director of the Rizzuli Institute at Bologna). The object of this operation is to use the voluntary movements of the muscles of the stumps to move the artificial limb (vitalize the prothesis). In the foreman, these flexors and extensors are covered separately by skin and project beyond the general surface of the stump. At the base of the two clubs thus formed are fastened celluloid rings which actuate chains and produce extension and flexion in the artificial hand. In an amputation of the lower part of the thigh a club loop is formed. By a special plastic operation a canal lined with skin is formed through the muscles and through this a rod is passed. A strap fastened to each end of this rod replaces the quadriceps tendon.

B. *Early Education of Muscles of Stumps.* Dr. Martin (Belgium) said that he begins movements in stumps on the day after the operation. After amputations in the upper extremity he prefers work with tools to mechano-therapy. This develops power of sensation in a stump. Earlier cooperation between surgeon and makers of artificial limbs is essential to greater success.

Dr. Ripert (France) reported 618 cases of *Partial Amputation of the Foot.* When an astragalectomy, subastragalian, or Lisfranc operation were not possible, Patil's advice should be followed and the foot sacrificed.

